# Childhood screening for type 1 diabetes comparing automated multiplex Antibody Detection by Agglutination-PCR (ADAP) with single plex islet autoantibody radiobinding assays

**DOI:** 10.1016/j.ebiom.2024.105144

**Published:** 2024-05-08

**Authors:** Alexander Lind, Eva Freyhult, Felipe de Jesus Cortez, Anita Ramelius, Rasmus Bennet, Peter V. Robinson, David Seftel, David Gebhart, Devangkumar Tandel, Marlena Maziarz, Helena Elding Larsson, Markus Lundgren, Annelie Carlsson, Anna-Lena Nilsson, Malin Fex, Carina Törn, Daniel Agardh, Cheng-ting Tsai, Åke Lernmark, the Better Diabetes Diagnosis (BDD) study group, Martina Persson, Gun Forsander, Johnny Ludvigsson, Ulf Samuelsson, Claude Marcus

**Affiliations:** a Department of Clinical Sciences, Lund University CRC, Malmö, Sweden; b Department of Cell and Molecular Biology, National Bioinformatics Infrastructure Sweden, Science for Life Laboratory, Uppsala University, Uppsala, Sweden; c Enable Biosciences Inc., South San Francisco, CA, USA; d Department of Clinical Sciences, Lund University, Lund, Sweden

**Keywords:** Type 1 diabetes, Insulin autoantibodies, GAD65 autoantibodies, IA-2 autoantibodies, ZnT8 autoantibodies, Radiobinding assay, Antibody detection by agglutination PCR, Diagnostic sensitivity, Diagnostic specificity

## Abstract

**Background:**

Two or more autoantibodies against either insulin (IAA), glutamic acid decarboxylase (GADA), islet antigen-2 (IA-2A) or zinc transporter 8 (ZnT8A) denote stage 1 (normoglycemia) or stage 2 (dysglycemia) type 1 diabetes prior to stage 3 type 1 diabetes. Automated multiplex Antibody Detection by Agglutination-PCR (ADAP) assays in two laboratories were compared to single plex radiobinding assays (RBA) to define threshold levels for diagnostic specificity and sensitivity.

**Methods:**

IAA, GADA, IA-2A and ZnT8A were analysed in 1504 (54% females) population based controls (PBC), 456 (55% females) doctor's office controls (DOC) and 535 (41% females) blood donor controls (BDC) as well as in 2300 (48% females) patients newly diagnosed (1–10 years of age) with stage 3 type 1 diabetes. The thresholds for autoantibody positivity were computed in 100 10-fold cross-validations to separate patients from controls either by maximizing the χ^2^-statistics (chisq) or using the 98th percentile of specificity (Spec98). Mean and 95% CI for threshold, sensitivity and specificity are presented.

**Findings:**

The ADAP ROC curves of the four autoantibodies showed comparable AUC in the two ADAP laboratories and were higher than RBA. Detection of two or more autoantibodies using chisq showed 0.97 (0.95, 0.99) sensitivity and 0.94 (0.91, 0.97) specificity in ADAP compared to 0.90 (0.88, 0.95) sensitivity and 0.97 (0.94, 0.98) specificity in RBA. Using Spec98, ADAP showed 0.92 (0.89, 0.95) sensitivity and 0.99 (0.98, 1.00) specificity compared to 0.89 (0.77, 0.86) sensitivity and 1.00 (0.99, 1.00) specificity in the RBA. The diagnostic sensitivity and specificity were higher in PBC compared to DOC and BDC.

**Interpretation:**

ADAP was comparable in two laboratories, both comparable to or better than RBA, to define threshold levels for two or more autoantibodies to stage type 1 diabetes.

**Funding:**

Supported by The Leona M. and Harry B. 10.13039/100007028Helmsley Charitable Trust (grant number 2009-04078), the Swedish Foundation for Strategic Research (Dnr IRC15-0067) and the 10.13039/501100004359Swedish Research Council, Strategic Research Area (Dnr 2009-1039). AL was supported by the DiaUnion collaborative study, co-financed by EU Interreg ÖKS, 10.13039/501100005275Capital Region of Denmark, Region Skåne and the Novo Nordisk Foundation.

**Research in context**

**Evidence before this study**

Screening for asymptomatic stage 1 and 2 type 1 diabetes is increasing. The gold standard radiobinding assay (RBA) that separate antibody bound from free labeled protein with Sepharose-Protein A does not lend itself to multiplex automated assays. There was a need to compare the automated multiplex Antibody Detection by Agglutination-PCR (ADAP) assay with the standard single plex RBA for threshold levels in order to delineate Stage 1 type 1 diabetes defined by the presence of two or more islet autoantibodies.

**Added value of this study**

The diagnostic specificity and sensitivity to fulfill the criteria for stage 1 type 1 diabetes was ascertained in two laboratories in the US and Sweden, ADAP robotic systems and compared to RBA. Almost 2500 samples from population based controls (PBC), obtained in the doctor's office controls (DOC) or from blood donor controls (BDC), were used to identify threshold levels for finding individuals in stage 1 type 1 diabetes. The findings add value to the worldwide effort to screen healthy populations of children in order to diagnose individuals in stage 1 type 1 diabetes and prepare for a subsequent clinical follow up.

**Implications of all the available evidence**

These findings should encourage studies of individuals who are either at increased genetic risk for type 1 diabetes or detected in screening efforts of children in the population to prevent ketoacidosis at stage 3 type 1 diabetes. Identification of children with stage 1 (normal blood glucose) or stage 2 (dysglycemia) type 1 diabetes has immediate implications to halt the progression to manifest diabetes by different interventions. The ADAP method is appropriate to large scale autoantibody screening and therefore important to both human health and to either slow or prevent progression to stage 3 type 1 diabetes.

## Introduction

Type 1 diabetes is caused by specific autoimmunity against the pancreatic islet beta cells, which are markedly reduced at the time of clinical diagnosis causing the need for life-long insulin replacement therapy.^1^^,^^2^ Standardized autoantibody tests are end-point measures of islet autoimmunity.^3^, ^4^, ^5^ The etiology of type 1 diabetes may involve environmental exposures that precede the appearance of a first autoantibody against either insulin (IAA) or glutamic acid decarboxylase (GADA).^6^ The subsequent pathogenesis is marked by IAA, GADA, or both, in addition to autoantibodies against islet antigen-2 (IA-2A) and zinc transporter 8 (ZnT8A).^2^^,^^7^ Two or more of these islet autoantibodies represent stage 1 (normal blood glucose) or stage 2 (abnormal glucose tolerance) type 1 diabetes.^8^ Clinical onset is stage 3 type 1 diabetes.^8^ Screening of either first degree relatives to patients with type 1 diabetes^9^^,^^10^ or of the general population^11^^,^^12^ allows the detection of individuals with multiple autoantibodies who have a defined increased risk for progression to clinical onset.^5^^,^^13^^,^^14^

There is a worldwide unmet need to screen for islet autoantibodies to prevent diabetes ketoacidosis^12^^,^^15^, ^16^, ^17^, ^18^ and as enrichment biomarkers in type 1 diabetes prevention trials, including registration studies, as endorsed by the European Medicines Agency.^19^ Population-based screening would be needed as only 10% of newly diagnosed type 1 diabetes children have a first degree relative with the disease.^7^^,^^8^^,^^11^^,^^20^ Autoantibody detection methods adapted to microliter volumes of sample, multiplexed to simultaneously detect all four islet autoantibodies with reliable precision and reproducibility would also be needed. The autoantibody biomarkers in type 1 diabetes have relied on RBA using *in vitro* transcription translation (ITT) to label autoantigens with e.g. ^35^S-methione and separation of free from antibody-bound autoantigens with protein A-Sepharose.^21^ The RBA, as a gold standard, is using either a WHO reference standard for GADA and IA-2A^22^ or a harmonized DK reference standard^23^ and is subject to regular standardization workshops.^24^ Prior attempts to replace the RBA with multiplex autoantibody assays include two-sided ELISA for GADA, IA-2A and ZnT8A,^25^^,^^26^ luciferase immunoprecipitation system (LIPS),^27^ multiplex assay by electrochemiluminescence (ECL)^28^ and the Antibody Detection by Agglutination-PCR (ADAP) assay.^29^, ^30^, ^31^, ^32^

Screening for stage 1 and 2 type 1 diabetes would entail that all four autoantibodies are analysed simultaneously in a small amount of blood obtained by sampling preferably at home. We have previously compared the automated multiplex ADAP assay with the gold standard RBA in 500 samples from patients newly diagnosed with type 1 diabetes and healthy controls.^30^ It was concluded that the simultaneous analysis of 5 different autoantibodies by ADAP in sample volume reduced to only 4 μL and at an increased lower detection limit makes the ADAP automated autoantibody assay advantageous for high throughput screening.^30^ The threshold levels are critical in any screening to control the false positive rate. Our analysis was therefore extended to 2300 patients, 1–10 years of age, with newly diagnosed stage 3 type 1 diabetes^7^^,^^33^^,^^34^ and 2495 controls to establish diagnostic accuracy by automated multiplex ADAP compared to standard RBA. Future screening efforts would require detailed analysis of threshold levels for positive samples to establish the diagnostic sensitivity of stage 1 type 1 diabetes.

The aim of the present study was therefore to compare the large number of samples previously analysed with standard RBA,^7^^,^^33^^,^^34^ also in healthy controls^20^^,^^35^ to identify possible heterogeneity in detecting autoantibodies in the multiplex automated ADAP and to compare assay performance in two different laboratories. We also wanted to analyse the most optimal diagnostic sensitivity and specificity for ADAP compared to RBA.

## Methods

### Healthy controls to define diagnostic specificity

The control samples were 1) 1504 PBC representing healthy children in the Celiac Prediction in Skåne (CiPiS) study,^20^^,^^35^ 2) 456 DOC representing healthy children from whom a blood sample had been obtained at a visit to a health care center or schools^36^, ^37^, ^38^ and 3) 535 BDC^39^^,^^40^ (Table 1).

**Table 1:** Summary of samples from population based controls (PBC), doctors office controls (DOC) blood donor controls (BDC) and patients with newly diagnosed Stage 3 type 1 diabetes (BDD).

	Population based controls (PBC)	Doctors office controls (DOC)	Blood donor controls (BDC)	Patients with new onset T1D (BDD)
n	1504	456	535	2300
Age (years) median (1st-3rd quartile)	10 (9–15)	13 (12–14)	46 (33–33)	7 (4–9)
Females	808 (54%)	252 (55%)	220 (41%)	1113 (48%)
EDTA plasma	1504 (100%)	2 (0.4%)	0 (0%)	72 (3.1%)
Serum	0 (0%)	454 (%)	535 (100%)	2228 (97%)
Sampling year				
2005	0 (0%)	0 (0%)	0 (0%)	185 (8.0%)
2006	0 (0%)	0 (0%)	0 (0%)	343 (15%)
2007	0 (0%)	0 (0%)	0 (0%)	347 (15%)
2008	0 (0%)	93 (20%)	59 (11%)	351 (15%)
2009	0 (0%)	179 (39%)	0 (0%)	407 (18%)
2010	73 (4.9%)	122 (27%)	0 (0%)	379 (16%)
2011	381 (25%)	62 (14%)	0 (0%)	0 (0%)
2012	363 (24%)	0 (0%)	0 (0%)	270 (12%)
2013	222 (15%)	0 (0%)	0 (0%)	18 (0.8%)
2014	5 (0.3%)	0 (0%)	0 (0%)	0 (0%)
2016	83 (5.5%)	0 (0%)	0 (0%)	0 (0%)
2017	151 (10%)	0 (0%)	0 (0%)	0 (0%)
2018	107 (7.1%)	0 (0%)	0 (0%)	0 (0%)
2019	102 (6.8%)	0 (0%)	476 (89%)	0 (0%)
2020	13 (0.9%)	0 (0%)	0 (0%)	0 (0%)

### Patients with type 1 diabetes to define diagnostic sensitivity

A total of 2300 incident children, 1–10 years of age, with newly diagnosed stage 3 type 1 diabetes, were selected from 4000 consecutively diagnosed 1–18 years old children, in the Swedish Better Diabetes Diagnosis (BDD) study.^7^^,^^36^ Blood samples were obtained either at diagnosis, before insulin was administered, or within 3 days after diagnosis (Table 1).

### GADA and IA-2A radiobinding assays

Recombinant GAD65 and IA-2 were labeled with ^35^S-methionine (PerkinElmer LifeSciences, Boston, MA, USA) by *in vitro* transcription translation in the TNT SP6 coupled reticulocyte lysate system (Promega, Southampton, U.K.).^7^^,^^21^ Full-length human GAD65 cDNA was subcloned into the pTNT vector (Promega) (pThGAD65).^41^ The intracellular domain (aa 603–980) of IA-2 was used in the IA-2ic cDNA (Michael Christie, School of Life Sciences, University of Lincoln, Lincoln, UK) initially cloned into the pSP64 Poly(A) vector (Promega)^42^ and then cloned by us into the pTNT vector. In both assays, duplicate of 2.5 μl serum samples were diluted in 60 μL of labeled antigen (about 25,000 cpm) diluted in Assay Buffer (150 mmol/L NaCl, 20 mmol/L TrisHCl (pH 7.4), 0.15% v/v Polysorbate 20, 0.1% w/v bovine serum albumin) in 96-well plates (Nunc V96 MicroWell™, Nunc A/S, Roskilde, Denmark). After incubation at 4 °C overnight on a plate shaker at 300 rpm, 50 μL were transferred to MultiScreen HTS-DV Plates (MilliporeSigma, Burlington, MA, USA) containing 50 μL 20% Protein A-Sepharose (Invitrogen, Waltham, MA, USA). The Protein A-Sepharose was washed three times in Assay Buffer at 4 °C the day before. The MultiScreen HTS-DV plates were coated overnight at +4 °C in Assay Buffer. The coating plates were washed six times in Washing Buffer (150 mmol/L NaCl, 20 mmol/L TrisHCl (pH 7.4) with 0.15% (v/v) Polysorbate 20) with a 405^TM^LS Microplate Strip Washer (Biotek Instruments, Inc. Winooski, VT, USA) and then dried for 5 min before 50 μL Optiphase Super mix scintillation cocktail was added to each well and the Sepharose-bound radioactivity counted in a 1450 MicroBeta® TriLux β-counter (PerkinElmer). GADA and IA-2A levels were expressed as units per milliliter (U/mL) derived from the WHO standard 97/550.^22^ Samples were considered positive if GADA levels were 34 U/mL and IA-2A levels 5 U/mL. The intra-assay CV for duplicates was 7% in the GADA and 11% in the IA-2A assay. In the Islet Autoantibody Standardization Program (IASP) 2020 workshop, our GADA assay had a workshop sensitivity of 64% and specificity of 98% and IA-2A with a workshop sensitivity of 72% and specificity of 100%. In the IASP 2023 workshop the sensitivity for GADA was 74% and the specificity 98% and the IA-2A sensitivity was 76% and specificity 100%.

### IAA radioimmunoassay

IAA was measured in radioimmunoassay as described^7^ using a two-step procedure.

*Noncompetitive step:* serum or plasma samples (7 microliter) in duplicate wells in a 96-well Nunc V96 MicroWell™ (Nunc A/S, Roskilde, Denmark) microplates, were incubated for 48 h at 4 °C on a shaker with 36 μL ^125^I-insulin (PerkinElmer, Boston, MA, USA) representing around 55,000 cpm/well. After incubation, 25 μL were transferred to MultiScreen HTS-DV Plates (MilliporeSigma, Burlington, MA, USA) containing 50 μl pre-washed 40% Protein A-Sepharose (Invitrogen, Waltham, MA, USA) in Assay Buffer. The radioactivity was measured in 1450 MicroBeta Tri-Lux Microplate Scintillation-Luminescence Counter (PerkinElmer).

*Competitive step:* samples found positive for IAA in the non-competitive assay were analysed with cold insulin to displace non-specifically bound ^125^I-insulin. Serum or plasma samples (7 μL) were added to four wells on a 96-well plate. ^125^I-insulin (36 μL 55,000 cpm/well) was added along with 0.072 IU (or 2 IU/mL) unlabeled insulin (Actrapid®; Novo Nordisk A/S, Bagsvaerd, Denmark) in the last two wells. The plates were incubated and examined under the same conditions as in the noncompetitive method. The reference standard in six-step, doubling dilution was an IAA high titre type 1 diabetes serum.^37^^,^^39^^,^^43^ IAA levels were calculated as relative in-house units and positivity for IAA was 0.79 relative units. The intra-assay CV was 6.0% and inter-assay 13.2%. In the IASP 2020 workshop, our IAA assay showed a workshop sensitivity of 20% and specificity of 100%. In the IASP 2023 workshop the sensitivity for IAA was 28% and the specificity 99%.

### ZnT8A radiobinding assays for three different epitopes

The serum samples were analysed for autoantibodies (ZnT8A) against arginine (ZnT8RA), tryptophan (ZnT8WA), and glutamine (ZnT8QA) on position 325 as described in detail.^7^^,^^44^ The cDNA clone (from John C. Hutton) with arginine was subjected to Phusion site-directed mutagenesis (Finnzymes Oy, Espoo, Finland) to prepare the tryptophan and glutamine variants. Duplicates of 5 μL serum or plasma diluted in 55 μL ^35^S-methionine-labeled antigen were incubated over night at 4 °C on a plate shaker at 300 rpm followed by protein A-Sepharose (Invitrogen, Waltham, MA, USA) incubation as described above. Antibody-bound radioactivity was counted in a 1450 MicroBeta Tri-Lux Microplate Scintillation-Luminescence Counter (PerkinElmer). Levels of autoantibodies were estimated from the in-house “3003” reference standard. The three ZnT8A assays showed comparable precision as intra-assay CV was 5.5% for ZnT8RA, 5.3% for ZnT8WA, and 4.9% for ZnT8QA and reproducibility as inter-assay CV was 13.8% for ZnT8RA, 6.7% for ZnT8WA, and 11.0% for ZnT8QA. In the IASP 2020 workshop, our laboratory showed a workshop sensitivity of 66% and specificity of 100% for ZnT8RA, 60% and 100% for ZnT8WA, and 42% and 100% for ZnT8QA, respectively. In IASP 2023 the workshop sensitivity was 54% and specificity 100% for ZnT8RA, 56% and 100% for ZnT8WA, and 44% and 100% for ZnT8QA, respectively.

### Antibody Detection by Agglutination-PCR (ADAP)

Previously, we have reported the 5-plex ADAP method for detection of four islet autoantibodies and tissue transglutaminase autoantibodies on an automated Hamilton MicroLab STAR systems.^30^ Briefly, 4 μL serum or plasma samples were incubated with 8 μL DNA-barcoded autoantigens at 37 °C for 30 min. Heparin plasma was avoided as heparin is a well-known inhibitor of the PCR. Tests have been carried out with up to 500 mg/dL of hemoglobin, 6.7 mg/dL of bilirubin and 3000 mg/dL of lipids and no interferences were observed. Autoantibodies present in the specimens would agglutinate the autoantigens into a dense immune-complex. Then, 4 μL of mixtures were aspired and mixed with 116 μL of ligation mixtures, where nearby DNA in the dense immune complex would be ligated to form a full-length DNA amplicon. Next, 25 μL of mixture above were further mixed with 25 μL PCR amplification mixtures containing primers for all 5 autoantibodies for a total of 13 PCR cycles using On Deck Thermocycler (ODTC, Inheco, Martinsried, Germany). The amplified product, 4 μL diluted in 76 μL double distilled water, was pipetted to 384 well plates containing the cognate primer pairs for each autoantibody to achieve specific quantification on a real-time quantitative PCR (RT-qPCR). At the Lund University CRC in Malmö, Sweden, the qPCR ready plates were transferred automatically to the Roche Light cycler 480 System II (Roche Diagnostics International AG, Rotkreuz, Switzerland) to enable a full sample-to-answer solution. It is also acknowledged that the present analysis of the 5-plex ADAP assays at the CRC laboratory included two different batches of reagents. In the first set of analyses the ZnT8A ADAP reagent were compromised and showed invalid results. The samples at CRC were re-analysed for ZnT8 autoantibodies with a new batch of ZnT8 ADAP reagent. In the 2023 IASP, the ADAP at Enable Biosciences had sensitivity /specificity of 90%/99% for GADA, 74%/100% for IA-2A, 62%/99% for IAA and 74%/100% for ZnT8A. The ADAP at Lund University CRC in Malmö had sensitivity /specificity of 90%/99% for GADA, 74%/99% for IA-2A, 42%/100% for IAA and 70%/100% for ZnT8A.

### Ethics

Separate written informed consents were obtained for all study participants in the Better Diabetes Diagnosis study. The Regional Ethics Board at Karolinska Institute approved the BDD study (Dnr 2004–826/1, 2006/1082–32, and 2009/1684–32). The studies of controls were approved by the Regional Ethical Review Board in Lund (Dnr LU 621-03, 2010/170 2013/541 and 2016/410) and all participants gave informed consent. The parents or guardians of under aged study participants gave informed consent.

### Statistical analysis

#### RBA values

The RBA derived concentration values equal to or below zero were imputed to minimum observed value for that particular autoantibody divided by two. The number of measurements below zero are reported in Supplemental Table S2. After imputation, values were log2-transformed before any further analyses. RBA value distributions are illustrated in density plots.

#### Duplicate samples analysed for intra-assay comparison

A total of 200 randomly selected patient samples were analysed twice in the ADAP assays located at various positions on the assay plates. The agreement between the two measurements was quantified by Pearson's correlation coefficient as well as summary statistics based on the paired difference (on log2-scale), the mean difference (bias) and standard deviation of differences (sddiff). In the following analyses only one measurement per sample was included.

#### Inter-laboratory comparison between the RBA and the ADAP assays

The Pearson correlations between RBA and ADAP at both Enable and CRC are computed for each of the five autoantibodies, both for all samples together and separately for patient and control samples. In addition, for each sample the difference between assays was computed and summary statistics computed; the mean difference and sddiff as a measure of the agreement between the two compared assays.

#### Comparison between patients and controls

The autoantibody levels were compared between patients and controls using t-tests. Furthermore, receiver operating characteristic (ROC) curves were used evaluate the diagnostic accuracy of the measured autoantibody levels. The area under the curve (AUC) of the ROC curve was computed for each autoantibody for both RBA and ADAP measurements using the R-package pROC.^45^ AUC confidence intervals are computed using DeLong's method.^46^ AUC between RBA and ADAP were compared using DeLong's test for paired samples as implemented in the pROC R-package.

#### RBA and ADAP thresholds

Both a selected threshold that give an optimal threshold (chisq) and 98% specificity (Spec98) was computed for separation of patients and controls. The thresholds for autoantibody positivity were computed in 100 balanced 10-fold cross-validations. Sensitivity and specificity were assessed on the test sets and summarized by mean and a 95% confidence interval estimated from the 1000 values.

### Role of funders

The Funders of this investigation has no role in study design, data collection, data analyses, interpretation, or writing of report.

## Results

### RBA value distribution

RBA threshold levels were based on the WHO reference standard for GADA and IA-2A and on in-house reference standards for IAA and ZnT8A (Supplemental Figure S1 and Supplemental Table S1). The differences in distribution between patient and control samples are indicated in density plots (Supplemental Figure S1). The increasing binding values in patient sera was not observed in the controls. Samples in the ZnT8A triple assay were not diluted. However, high level samples in the ZnT8RA, ZnT8WA and ZnT8QA assays were diluted to read the values off the linear portion of the respective standard curve.

### Inter-laboratory comparison between the RBA and the ADAP assays

**Fig. 1:**
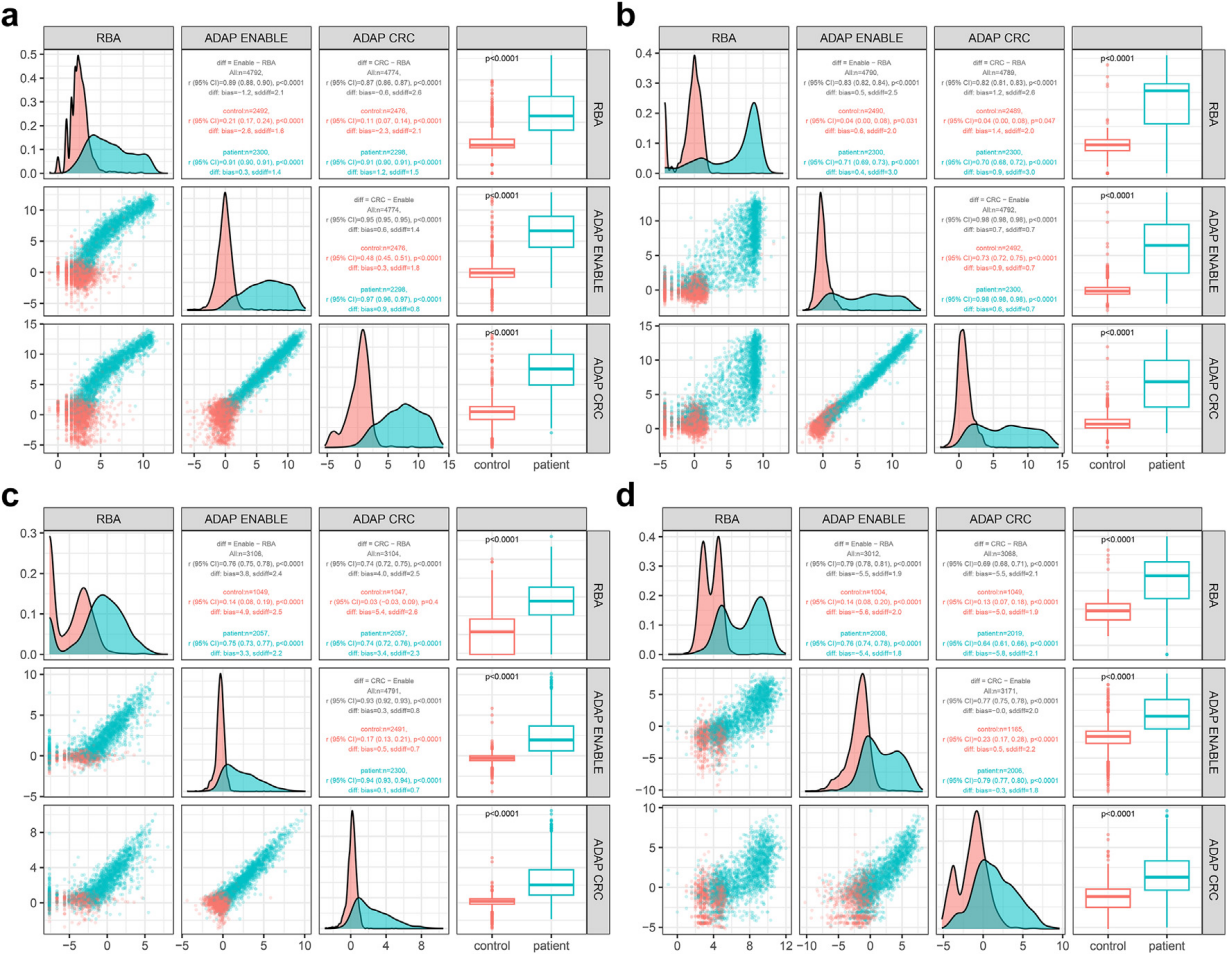
Distribution of autoantibody levels and correlations between RBA and the two ADAP analyses at Enable and CRC, respectively. The correlation coefficients are shown separately for all controls (red) and patients with stage 3 type 1 diabetes (blue). Correlation coefficients over all as well as for patients (blue) and controls (red) are shown. The distributions are shown for GADA (panel a), IA-2A (panel b), IAA (panel c) and ZnT8A (panel d). The difference between controls and patients are also shown with p-values in a Welch t-test. ∗∗∗ indicates a p-value <0.001.

The distributions of autoantibody levels and correlations between RBA and ADAP at both the Enable and CRC laboratories are shown for GADA (Fig. 1a), IA-2A (Fig. 1b), IAA (Fig. 1c) and ZnT8A (Fig. 1d). Comparing all samples showed significant correlations between the two assays for all four autoantibodies, in particular for the patient samples (Welch’s t-test). The inter-assay variability shown by Pearson's correlation (r), 95% Confidence Intervals (95% CI), average mean difference between the assays (bias) and the sddiff are detailed in panel a for GADA, panel b for IA-2A, panel c for IAA and panel d for ZnT8A (Fig. 1). While the US (Enable) and Sweden (CRC) laboratories did not differ, variability and outliers were detected when the ADAP assays were compared to the individual RBA autoantibody analyses.

The measured levels of GADA, IA-2A, IAA and ZnT8A were also compared between the three control groups, PBC, DOC and BDC in the two ADAP assays, CRC and Enable as well as with the RBA (Supplemental Figure S2). The PBC tended to be associated with lower levels in the two ADAP assays. The RBA showed more variable levels in both PBC, DOC and BDC.

### Duplicate samples analysed for intra-assay comparison

A total of 200 randomly selected samples were analysed twice with the ADAP assays with samples placed at various positions on the assay plates to reduce the potential source of technical bias. Bland–Altman plotsshowing the correspondence between the replicate values are shown in Supplemental Figure S3. The agreement between the two measurements was quantified by Pearson's correlation coefficient, the sddiff between the two measurements (Supplemental Figure S3).

### RBA and ADAP ROC curves

**Fig. 2:**
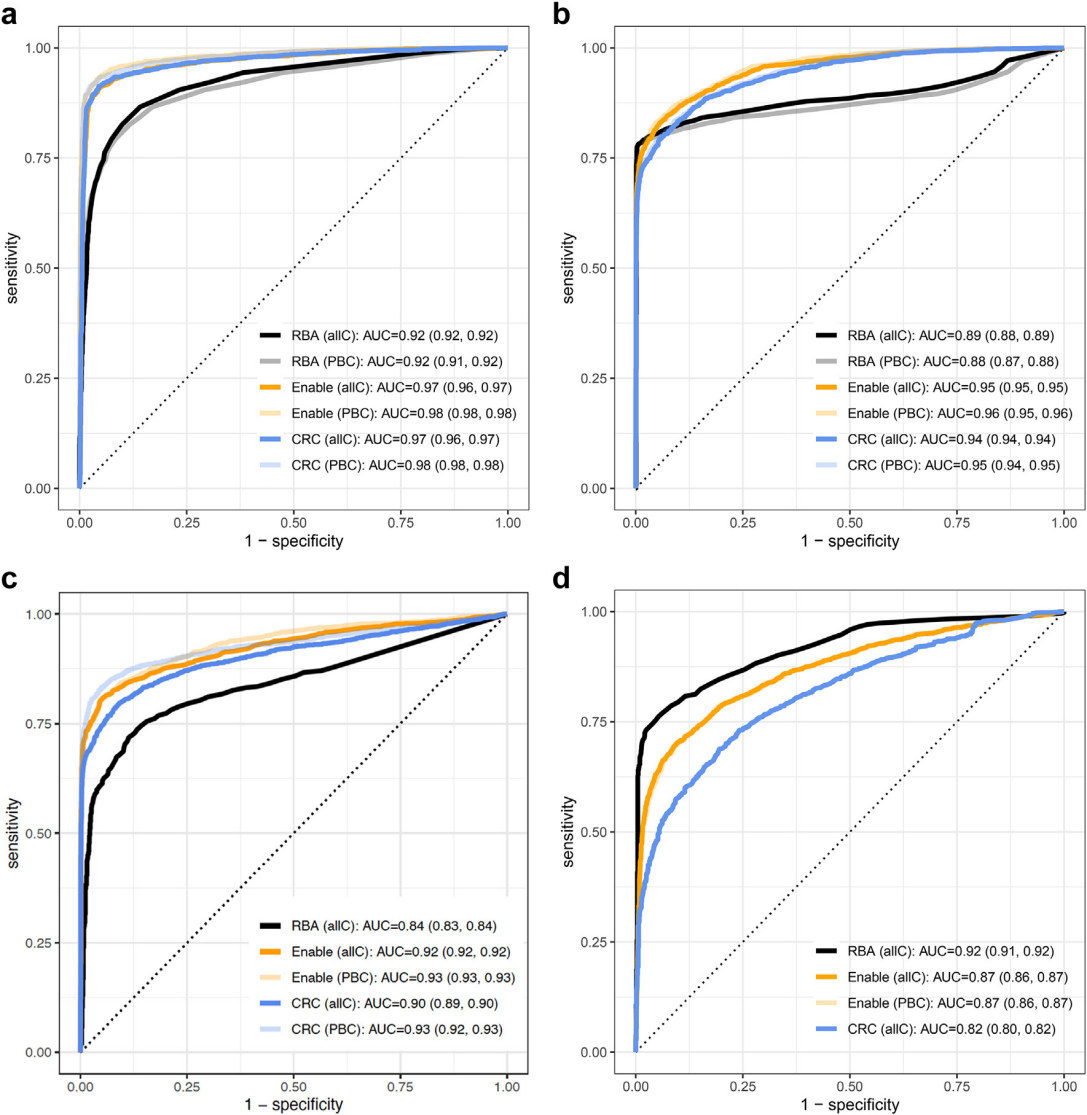
ROC curves and AUC with 95% CI (within parenthesis) values for the four islet autoantibodies in RBA (black) and ADAP at Enable (orange) as well as CRC (blue). The ROC curves and AUC values when PBC only were used are shown in light colours. Panel a. ROC curves for GADA. While there was no difference between ADAP at Enable and CRC, respectively, the AUC was different to RBA. Panel b. ROC curves for IA-2A. While there was a slight difference between ADAP at Enable and CRC, the AUC for the RBA assay was smaller. Panel c. ROC curves for IAA. There was a slight difference between ADAP at Enable and CRC. The AUCs for the ADAP assays were larger compared to that for the RBA. Panel d. ROC curves for ZnT8A. The RBA showed the best performance with the AUC larger than that for ADAP Enable and ADAP CRC. It is noted that the RBA is carried out with all three variants of the position 325 amino acids R, W, and Q. The ADAP assay is analysing the R and W variants.

The AUC of the ROC curves were analysed for each autoantibody in the two ADAP assays and compared to RBA (Fig. 2). GADA was superimposable between the two laboratories (Fig. 2a) while the RBA AUC was statistically significantly smaller compared with ADAP AUC (Table 2). IA-2A was characterized by RBA samples being negative or low at a high frequency resulting in a sharp inflection point in the ROC curve (Fig. 2b). The two ADAP assays showed similarly shaped ROC curves and both were statistically significantly higher compared to that for RBA (Table 2). Similarly for IAA, the two ADAP ROC curves were higher compared to that for the RBA (Fig. 2c). The IAA ROC AUC in ADAP had a significantly better performance in the US compared to the Sweden laboratory. The RBA ROC AUC was lower compared to both ADAP assays (Fig. 2c; Table 2). For the triple ZnT8A, the RBA assay performed better than either of the ADAP assays, and ADAP at the US performed better than ADAP in the Swedish laboratory as evaluated using the ROC (Fig. 2d) and AUC (Table 2).

**Table 2:** Comparison between all ROC curves AUC for each autoantibody and combination between RBA and ADAP at CRC or Enable, respectively.

AAb	Assay 1	Assay 2	n	AUC1	AUC2	AUC diff (95% CI)	p-value
GADA	Enable	CRC	4774	0.97	0.97	−0.00 (−0.00, 0.00)	0.71
GADA	RBA	CRC	4774	0.92	0.97	−0.05 (−0.05, −0.04)	<0.0001
GADA	RBA	Enable	4792	0.92	0.97	−0.04 (−0.05, −0.04)	<0.0001
IA-2A	Enable	CRC	4792	0.95	0.94	0.01 (0.01, 0.02)	<0.0001
IA-2A	RBA	CRC	4789	0.89	0.94	−0.05 (−0.06, −0.04)	<0.0001
IA-2A	RBA	Enable	4790	0.89	0.95	−0.06 (−0.07, −0.05)	<0.0001
IAA	Enable	CRC	4791	0.92	0.90	0.02 (0.01, 0.03)	<0.0001
IAA	RBA	CRC	3104	0.84	0.86	−0.02 (−0.04, −0.01)	0.0011
IAA	RBA	Enable	3106	0.84	0.91	−0.07 (−0.08, −0.05)	<0.0001
ZnT8A	Enable	CRC	3171	0.87	0.82	0.06 (0.04, 0.07)	<0.0001
ZnT8A	RBA	CRC	3068	0.92	0.82	0.10 (0.08, 0.12)	<0.0001
ZnT8A	RBA	Enable	3012	0.92	0.87	0.05 (0.03, 0.06)	<0.0001

ROC curves are computed for the separation of patients with stage 3 type 1 diabetes and all controls.

AAb is autoantibody.

### RBA and ADAP optimal thresholds for staging type 1 diabetes

Optimal threshold was computed based on 100 10-fold cross-validations. First, an optimal threshold is computed to maximize the χ^2^-statistic for separating the BDD type 1 diabetes patients from controls (chisq). Second, a threshold that would give 98% specificity (spec98) for all autoantibodies was selected. Mean values and 95% CI for threshold, sensitivity and specificity as computed based on the 1000 test sets from the 10-fold cross-validations either using all controls (Table 3) or the PBC (Table 3). The two ADAP analyses compare favorably with the RBA assays, while the RBA assays tended to have lower diagnostic specificity.

**Table 3:** Optimal thresholds computed based on 100 10-fold cross-validations.

a) Thresholds evaluated on the BDD patients and all controls.				
Autoantibody	Assay	Threshold	Sensitivity	Specificity
**chisq**				
GADA	CRC	2.42 (2.42, 2.43)	0.91 (0.87, 0.94)	0.96 (0.93, 0.98)
GADA	Enable	1.97 (1.65, 1.98)	0.90 (0.85, 0.93)	0.96 (0.93, 0.98)
GADA	RBA	3.70 (3.70, 3.81)	0.82 (0.77, 0.87)	0.90 (0.86, 0.94)
IA-2A	CRC	2.74 (2.70, 2.74)	0.79 (0.74, 0.84)	0.94 (0.92, 0.97)
IA-2A	Enable	1.55 (1.49, 1.66)	0.82 (0.77, 0.87)	0.95 (0.92, 0.98)
IA-2A	RBA	1.93 (1.89, 2.00)	0.78 (0.73, 0.83)	0.99 (0.98, 1.00)
IAA	CRC	0.82 (0.79, 0.95)	0.76 (0.69, 0.82)	0.94 (0.90, 0.97)
IAA	Enable	0.42 (0.40, 0.42)	0.80 (0.75, 0.85)	0.95 (0.92, 0.98)
IAA	RBA	−2.06 (−2.40, −2.06)	0.73 (0.67, 0.79)	0.87 (0.79, 0.94)
ZnT8A	CRC	0.07 (−0.19, 0.81)	0.65 (0.52, 0.76)	0.82 (0.70, 0.93)
ZnT8A	Enable	0.25 (0.23, 0.25)	0.66 (0.60, 0.72)	0.94 (0.91, 0.96)
ZnT8A	RBA	5.13 (5.00, 5.32)	0.76 (0.70, 0.81)	0.94 (0.88, 0.99)
***spec98***				
GADA	CRC	3.18 (3.13, 3.24)	0.87 (0.83, 0.91)	0.98 (0.96, 1.00)
GADA	Enable	2.59 (2.36, 2.69)	0.86 (0.82, 0.90)	0.98 (0.96, 1.00)
GADA	RBA	5.25 (5.04, 5.39)	0.58 (0.51, 0.64)	0.98 (0.96, 1.00)
IA-2A	CRC	3.40 (3.35, 3.42)	0.73 (0.68, 0.79)	0.98 (0.96, 1.00)
IA-2A	Enable	2.16 (2.09, 2.19)	0.77 (0.72, 0.82)	0.98 (0.96, 1.00)
IA-2A	RBA	1.68 (1.63, 1.68)	0.79 (0.74, 0.84)	0.98 (0.96, 1.00)
IAA	CRC	1.10 (1.08, 1.11)	0.69 (0.63, 0.75)	0.98 (0.96, 1.00)
IAA	Enable	0.65 (0.64, 0.67)	0.74 (0.69, 0.80)	0.98 (0.96, 1.00)
IAA	RBA	−0.33 (−0.66, −0.20)	0.47 (0.40, 0.55)	0.98 (0.95, 1.00)
ZnT8A	CRC	2.18 (2.07, 2.21)	0.38 (0.31, 0.44)	0.98 (0.95, 1.00)
ZnT8A	Enable	1.21 (1.12, 1.35)	0.53 (0.47, 0.59)	0.98 (0.96, 1.00)
ZnT8A	RBA	5.43 (5.32, 5.46)	0.72 (0.66, 0.77)	0.98 (0.94, 1.00)

b) Thresholds evaluated on the BDD patients and PBC				
Autoantibody	Assay	Threshold	Sensitivity	Specificity
**chisq**				
GADA	CRC	2.12 (2.09, 2.42)	0.93 (0.89, 0.96)	0.95 (0.92, 0.99)
GADA	Enable	1.20 (0.92, 1.33)	0.93 (0.90, 0.96)	0.95 (0.89, 0.98)
GADA	RBA	3.72 (3.46, 3.91)	0.82 (0.76, 0.88)	0.88 (0.80, 0.94)
IA-2A	CRC	2.18 (1.74, 2.74)	0.84 (0.77, 0.90)	0.89 (0.80, 0.96)
IA-2A	Enable	1.25 (1.04, 1.49)	0.85 (0.79, 0.90)	0.92 (0.86, 0.98)
IA-2A	RBA	1.93 (1.89, 2.00)	0.78 (0.73, 0.83)	0.99 (0.98, 1.00)
IAA	CRC	0.60 (0.53, 0.61)	0.83 (0.78, 0.88)	0.95 (0.90, 0.98)
IAA	Enable	0.39 (0.23, 0.42)	0.81 (0.76, 0.85)	0.94 (0.88, 0.98)
IAA	RBA	−1.25 (−2.06, −1.12)	0.60 (0.53, 0.73)	0.93 (0.43, 1.00)
ZnT8A	CRC	−3.39 (−3.50, −1.48)	0.97 (0.88, 0.99)	0.30 (0.13, 0.55)
ZnT8A	Enable	−0.03 (−0.05, 0.25)	0.69 (0.63, 0.75)	0.90 (0.85, 0.95)
ZnT8A	RBA	4.46 (4.46, 4.46)	0.89 (0.85, 0.93)	0.69 (0.25, 1.00)
***spec98***				
GADA	CRC	2.57 (2.46, 2.62)	0.90 (0.86, 0.94)	0.98 (0.95, 1.00)
GADA	Enable	1.86 (1.81, 1.90)	0.90 (0.86, 0.93)	0.98 (0.96, 1.00)
GADA	RBA	5.01 (4.81, 5.11)	0.61 (0.55, 0.67)	0.98 (0.95, 1.00)
IA-2A	CRC	3.31 (3.28, 3.34)	0.74 (0.69, 0.79)	0.98 (0.95, 1.00)
IA-2A	Enable	2.05 (1.98, 2.09)	0.78 (0.73, 0.83)	0.98 (0.95, 1.00)
IA-2A	RBA	1.74 (1.72, 1.77)	0.79 (0.74, 0.83)	0.98 (0.95, 1.00)
IAA	CRC	0.78 (0.74, 0.80)	0.78 (0.73, 0.84)	0.98 (0.95, 1.00)
IAA	Enable	0.67 (0.64, 0.68)	0.74 (0.68, 0.79)	0.98 (0.95, 1.00)
IAA	RBA	−1.07 (−1.20, −1.06)	0.58 (0.51, 0.65)	0.98 (0.80, 1.00)
ZnT8A	CRC	2.05 (1.89, 2.09)	0.39 (0.32, 0.46)	0.98 (0.89, 1.00)
ZnT8A	Enable	1.34 (1.21, 1.42)	0.52 (0.46, 0.58)	0.98 (0.95, 1.00)
ZnT8A	RBA	5.79 (5.49, 5.82)	0.66 (0.60, 0.74)	0.97 (0.75, 1.00)

Mean and 95% CI for threshold, sensitivity and specificity is computed based on the 1000 test sets from the 10-fold cross-validations.

**Fig. 3:**
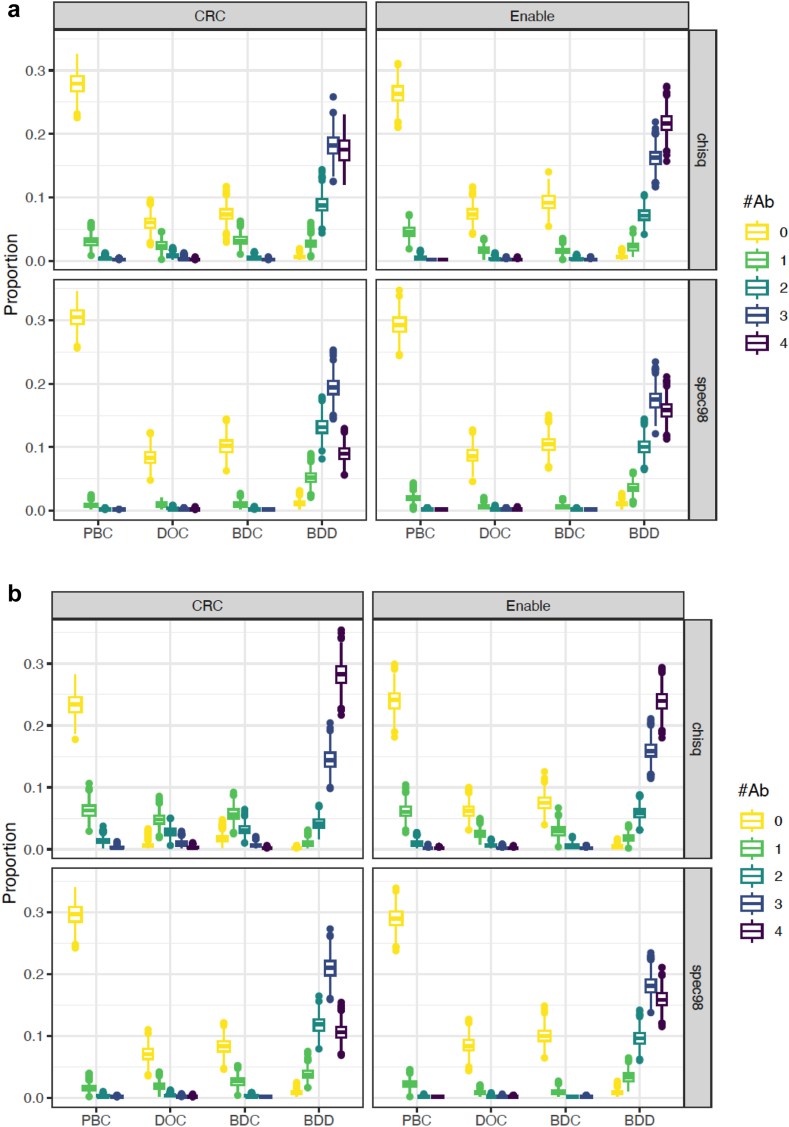
Proportion of samples positive for 0–4 (GADA, IA-2A, IAA and ZnT8A) islet autoantibodies. The proportion is based on the optimal threshold (chisq or spec98) for CRC and Enable, respectively, and computed for each of the 1000 test sets in the 10-fold cross-validations shown as boxplots. The colour code for the number of islet autoantibodies is shown. Panel a. Thresholds computed based on all controls and BDD type 1 diabetes patients. Panel b. Thresholds computed based on PBC and BDD type 1 diabetes patients.

The proportion of positive autoantibodies among the PBC, DOC and BDC as well as the BDD patients were analyzed using thresholds defined by chisq or Spec98 in all controls and BDD patients at either the CRC in Sweden or Enable in the US (Fig. 3, panel a). The proportions differ when the thresholds were based on the PBC and all BDD patients comparing CRC with Enable (Fig. 3, panel b).

Sensitivity and specificity to detect stage 1 or stage 2 type 1 diabetes as defined by at least two autoantibodies (AAb2) was compared to one (AAb1) positive autoantibody in the two ADAP assays and RBA (Supplemental Table S3). Mean and 95% CI for sensitivity and specificity for both chisq and Spec98 was computed based on the 1000 test sets from the 10-fold cross-validations. The number of positive autoantibodies using either all controls (Supplemental Table S3a) or PBC only (Supplemental Table S3b) for one autoantibody or more (1 AAb) showed reduced specificity for stage 3 type 1 diabetes regardless whether all controls or PBC were used as controls. Two or more autoantibodies restored the specificity for a stage 1 as well as for stage 3 type 1 diabetes diagnosis. PBC (children) as controls also showed higher sensitivity in all three assays compared to BDC (adult blood donors) as controls.

## Discussion

The aim to analyse a large number of samples to identify possible heterogeneity in detecting autoantibodies in a multiplex automated ADAP compared to single plex RBA showed minor variability between the two ADAP assays but a greater difference to RBA diagnostic performance. A major finding in this comprehensive comparison between gold standard RBA and ADAP was that the two ADAP assays performed in two different laboratories in the US and Sweden, respectively, were comparable when analyzing the samples on two different custom-modified fully automated Hamilton MicroLab STAR® instruments.

First, the comparison between the two automated ADAP analyses showed that the ADAP system is robust as there was a remarkable similarity in the GADA analysis and only limited differences in the assays for IA-2A and IAA. The differences observed in the two instruments may be explained by differences in handling of the samples prior to the automated pipetting in the two different instruments as well as the final PCR step. All ADAP analyses across the two laboratories were conducted based on split aliquots of the samples.

Second, the better diagnostic performances in the ADAP assays compared to gold standard RBA may be due to the vastly different approach to detect the autoantibody bound antigen. The RBA has since 1994^21^ used Protein A-Sepharose to separate free from autoantibody bound labeled autoantigen. Protein A has a high affinity for IgG1, IgG2 and IgG4 but not for IgG3 and IgM. The ADAP, based on proximity ligation between the oligonucleotides conjugated to the autoantigen may agglutinate all subtypes of IgG as well as IgM. It was therefore hypothesized that ADAP may have a higher diagnostic sensitivity and specificity than the RBA. The analysis of IAA by ADAP may contribute to an improved sensitivity as IAA tends to be the first appearing autoantibody in screening infants and young children for islet autoimmunity.^47^

Third, in only one instance the RBA performed better than the ADAP assay. The ZnT8A RBA represents a triple analysis as it incorporates all three variants, ZnT8R, ZnT8W and ZnT8Q at amino acid residue position 325.^7^ The design of the triple ZnT8A RBA assay was to detect autoantibodies to all three variants in a screening approach and then dissect the ZnT8 autoantibody appearance by analyzing all three subtypes individually to discern which ZnT8A was unique. The difference in ADAP between Enable and CRC is likely to primarily be explained by differences in the batch of reagents used to detect ZnT8A. The CRC analysed the ZnT8A fourth months apart from Enable with a different reagent batch. The difference between ADAP and RBA to detect ZnT8A may be due to the triple design in the RBA.^7^^,^^44^ The contribution of ZnT8QA to type 1 diabetes pathogenesis is often questioned. In two independent clinical trials it was observed that ZnT8QA was associated with impaired glucose metabolism.^48^^,^^49^ Also, residual beta-cell function was higher in patients with stage 3 type 1 diabetes who did not experience decreasing levels of ZnT8QA after diagnosis.^50^ Further studies will be needed to explore the three different variants of ZnT8A in the risk for stage 1 and 2 type 1 diabetes.

The statistical analyses of the reciprocal comparisons showed significant correlations between all three assays indicating acceptable performance of the ADAP technology. The highly significant correlations observed when either all samples or the patients only, but not the controls only, were compared is supporting the notion that the RBA and ADAP assay formats are similar. The low correlations between measurements for control samples are expected as the control samples have very low levels or no autoantibodies (the values are more or less zero, Supplemental Table S2). It was indeed possible to conclude that the PBC differed from the DOC and BDC. The PBC, randomly selected from the childhood population^20^ had lower levels of autoantibodies than DOC and BDC. A possible explanation that needs further studies was that the PBC were plasma, not serum, samples. The normal range will be easier to define and after the threshold (also known as cut point or cutoff) will be defined to avoid false negatives but allow false positives to be sorted out in subsequent follow up samples to identify true positives. In the ADAP assay, ΔCt values are obtained through a PCR reaction that is run for up to 40 cycles to detect low level autoantibodies. The RBA on the other hand has low level cpm for negative sera (resulting in poor intra-assay coefficient of variation) and high level cpm for positive samples. The RBA is clearly not normally distributed and all statistical analysis were carried out on log2 values.

Another difference between RBA and ADAP is that the ΔCt values were taken as a direct measure without the use of a calibration curve. All RBA values were obtained after each unknown sample was read off the respective calibration or standard curve. It is well known that the RBA assay requires calibration standard curves^22^^,^^23^ to reduce both inter-assay and inter-laboratory variation. It makes the single plex RBA both cumbersome and demanding in any screening approach of the population or of large number of first-degree relatives. The ADAP is multiplex and requires only 4 μL serum or plasma and would therefore seem to be the first line method to screen with the possibility that RBA or similar methods would be used for confirmation or validation. Other single or multiplex assays to consider include two-sided ELISA for GADA, IA-2A and ZnT8A,^25^^,^^26^ luciferase immunoprecipitation system,^27^ and multiplex assay by electrochemiluminescence.^28^ In these assays, the cost per sample per autoantibody is comparable to ADAP.

A limitation to the 4-plex ADAP analysed on the fully automated Hamilton MicroLab STAR® instrument is the 6–7 h required for a run. Previously reported Hamilton MicroLab STAR® with a single manual qPCR plate transfer step could reduce the run to 2–3 h.^30^ This is comparable to two-side ELISA and provides results for multiple autoantibodies from a single run. Such version of the ADAP assay may be suitable when rapid results are needed for decision making, such as the use of pancreas for transplantation or research in nPOD^25^ or the Nordic Network for Islet Transplantation.^51^ Further studies are needed to further explore the possibility of developing the ADAP assay for point-of-care use with results within an hour.

The staging of type 1 diabetes^8^ has provided an incitement to screen the general population as well as first degree relatives to patients with type 1 diabetes for islet autoantibodies^11^^,^^12^^,^^52^ to identify individuals at increased risk for either stage 2 or stage 3 type 1 diabetes. Stage 1 is defined as two or more autoantibodies and this has been taken into account in our analysis. Traditionally, threshold levels in assays with data that are normally distributed are set at a mean value plus two or three standard deviations. However, autoantibody index^21^ or harmonized (WHO or DK standard)^22^^,^^23^ units are not normally distributed. The smallest and largest measurements may differ by at least an order of magnitude in both patients and control individuals. ROC curves to establish threshold may not always provide the most effective threshold, and the percentile distribution among control individuals needs to be accounted for.

An alternative approach to set the specificity and sensitivity thresholds for IAA, GADA, IA-2A and ZnT8A was to normalize values and bring them onto a comparable scale such as the natural logarithm or log scale. The ordered autoantibody measurements were plotted against the quantiles of a standard normal distribution in both patients and control individuals^21^^,^^53^ A threshold for autoantibody status were determined graphically from the quantile–quantile plots for patients and control subjects. The threshold was defined by inspecting the vertical axis describing the ordered autoantibody measurements for the lowest value at which the slope of the line within the plot changed in both patients and control individuals. Inspecting the vertical line allow diagnostic specificity to be defined at the most effective percentile of the control population. The fact that the threshold level has a confidence interval is rarely taken into account. In the present study, we performed 100 10-fold cross-validations to compute the thresholds for autoantibody positivity. Thresholds may be presented as median and IQR based on the cross-validations and diagnostic sensitivity and specificity are computed for the test sets. Median and IQR were presented (Fig. 1) or as mean with 95th percentile (Table 3, Supplemental Table S3). In screening the population for islet autoantibodies, it may be recommended to use the lower 95th percentile as threshold to allow low level or false positive samples (not to miss anyone in the first screen) to be subjected to confirmatory analysis. The upper 95th percentile or the mean threshold level may be used to exclude false positives.

The difference in sensitivity, but not in specificity, for spec 98 threshold when comparing PBC and BDC as controls may have several explanations. The BDC samples are all from adult blood donors while the PBC were collected from children (Table 1). The time in storage and use differ. The PBC were all plasma while the BDC samples were serum. The data indicate that the selection of controls sampled at the same time and being matched to children without or with increased genetic risk for type 1 diabetes will be needed in future attempt to screen for stage 1 and 2 type 1 diabetes.

Taken together, the similarities between RBA and ADAP measurements are promising enough to take advantage of unique features of the ADAP technology. First, the low volume (4 microliter) of serum or plasma (heparin plasma needs to be avoided as heparin interferes with the PCR reaction). Second, detection of antibodies to multiple antigens, not only autoantigens but also of virus antigens.^54^ Third, there is a worldwide effort to limit the use of radioactivity. Finally, while the RBA is dependent on IgG binding proteins (Protein A and Protein G), the ADAP is likely to detect any antibody that is able to bring the oligonucleotide labelled antigens in close proximity for hybridization and subsequent PCR. The present comprehensive analysis of three different control groups and newly diagnosed stage 3 type 1 diabetes children to define threshold and thereby the normal range as well as diagnostic specificity and sensitivity should encourage population-based screening as well as screening of individuals who are at increased genetic risk for type 1 diabetes to prevent ketoacidosis at the clinical onset of type 1 diabetes.^52^ The presence of single autoantibody positivity in the three control populations in this cross-sectional observational study may represents individuals with either persistent autoantibody at risk for stage 3 type 1 diabetes (15/100 over 10 years), persistent autoantibody at risk for a second autoantibody and stage 1 type 1 diabetes or simply reverting autoantibodies.^55^ The screening effort also allow the identification of stage 1 individuals to develop methods to prevent them from progressing to stage 2. The screening effort should also include stage 2 type 1 diabetes individuals who would be eligible to life style intervention^56^ or FDA approved Teplizumab treatment^57^ to delay the onset of stage 3 type 1 diabetes.

## Contributors

These authors contributed equally: Alexander Lind, Eva Freyhult and Felipe de Jesus Cortez.

The study was designed by Å.L., A.L., A.R., C.T.T., P.V.R. and D.S. Samples were provided by H.E.L., M.L., A.C., A.L.N., M.F., C.T., and D.A. and analysed by A.L., A.R., F.J.C., R.B., D.G., D.T. and M.M. The data was organized and analysed by E.F., A.L., A.R. and F.J.C. Å.L. and C.T.T. wrote the first draft. A.L., E.F., A.R., C.T.T. and Å.L. have verified the underlying data. All authors reviewed and edited the manuscript and approved it for submission.

Members of the Better Diabetes Diagnosis (BDD) study group are listed in the Supplemental Group Authors List file.

## Data sharing statement

Data used in this article is available from the authors upon request.

Details of the database and the code used is available from the authors upon request as outlined by the National Bioinformatics Infrastructure Sweden.

## Declaration of interests

FJC, DG, DT, PVR, DS and CTT are employed by Enable Biosciences. FJC, DG, DT, PVR, DS and CTT are shareholders of Enable Biosciences. PVR and CTT are inventors of the ADAP patent licensed from University of California, Berkeley to Enable Biosciences. This does not alter our adherence to journal policies on sharing data and materials. All authors critically reviewed and approved the manuscript.

## Appendix A Supplementary data

Supplemental Better Diabetes Diagnosis (BDD) Study Group
