# Supplementary material for: Childhood screening for type 1 diabetes comparing automated multiplex Antibody Detection by Agglutination-PCR (ADAP) with single plex islet autoantibody radiobinding assays
Source: eBioMedicine. 2024 May 8;104:105144. doi: 10.1016/j.ebiom.2024.105144 (PMC11090024; doi:10.1016/j.ebiom.2024.105144)
Supplement: Supplemental Better Diabetes Diagnosis (BDD) Study Group [file mmc1.docx]

**Supplemental Better Diabetes Diagnosis (BDD) study group**

Members of the Better Diabetes Diagnosis (BDD) study group are

First name Surname

Martina Persson ^a^

Gun Forsander ^b^

Johnny Ludvigsson ^c^

Ulf Samuelsson ^c^

Claude Marcus ^d^

^a^Department of Medicine, Clinical Epidemiology, Karolinska University Hospital, Stockholm, Sweden

^b^ Deptartment of Pediatrics, Institute for Clinical Sciences, Sahlgrenska Academy, University of Gothenburg and the Queen Silvia Childrens Hospital, Sahlgrenska University Hospital, Gothenburg, Sweden

^c^ Crown Princess Victoria Children´s Hospital and Division of Pediatrics, Deptarment of Biomedical and Clinical Sciences, Linköping university, Sweden

^d^ Department of Clinical Science, Karolinska Institutet, Huddinge, Sweden
