# Supplementary material for: Childhood screening for type 1 diabetes comparing automated multiplex Antibody Detection by Agglutination-PCR (ADAP) with single plex islet autoantibody radiobinding assays
Source: eBioMedicine. 2024 May 8;104:105144. doi: 10.1016/j.ebiom.2024.105144 (PMC11090024; doi:10.1016/j.ebiom.2024.105144)
Supplement: Supplemental Tables S1–S7 [file mmc2.docx]

**Supplemental Tables 1-7**

***Supplemental Table 1.*** RBA normal thresholds (original scale, not log2-scale) in units/mL.

Autoantibody Threshold n Sensitivity (%) Specificity (%) PPV NPV

GADA 34.00 4793 60.35 97.87 96.32 72.79

IA-2A 5.00 4791 76.61 99.68 99.55 82.19

IAA 0.79 3106 46.48 98.00 97.85 48.29

ZnT8A NA 3070 55.02 99.43 99.46 53.43

NA is not applicable. PPV and NPV are positive and negative predictive value, respectively. ZnT8A was calculated by using the threshold in the triple ZnT8A with the additional requirement that at least one of the individual ZnT8RA, ZnT8WA or ZnT8QA should be above their respective threshold.

***Supplemental Table 2A:*** Optimal threshold based on the random selection of 10% of all individuals using the 98% specificity (spec98) for finding the optimal threshold. The data is based on the population based controls (PBC) and stage 3 type 1 diabetes individuals.

**Autoantibody Assay Threshold n Sensitivity (%) Specificity (%) PPV NPV**

GADA CRC 2.49 380 93.48 98.00 98.62 90.74

GADA Enable 1.66 380 92.61 98.00 98.61 89.63

GADA RBA 4.39 380 73.04 98.00 98.25 70.33

IA-2A CRC 3.14 381 76.96 98.01 98.33 73.63

IA-2A Enable 1.78 381 83.48 98.01 98.46 79.57

IA-2A RBA 1.70 380 77.83 98.67 98.90 74.37

IAA CRC 0.87 381 72.61 98.01 98.24 70.14

IAA Enable 0.63 381 75.22 98.01 98.30 72.20

IAA RBA 1.37 210 24.02 100.00 100.00 3.73

ZnT8A CRC 3.95 220 17.68 100.00 100.00 11.89

ZnT8A Enable 1.15 370 51.98 97.90 97.52 56.22

ZnT8A RBA 8.34 204 37.37 100.00 100.00 4.62

***Supplemental Table 2B*:** Optimal threshold based on the random selection of 10% of all individuals using the Chi^2^ metric for finding the optimal threshold. The data is based on the PBC and stage 3 type 1 diabetes individuals.

**Autoantibody Assay Threshold n Sensitivity (%) Specificity (%) PPV NPV**

GADA CRC 2.44 380 94.78 98.00 98.64 92.45

GADA Enable 0.81 380 97.39 94.67 96.55 95.95

GADA RBA 3.70 380 87.39 91.33 93.93 82.53

IA-2A CRC 2.27 381 87.83 92.05 94.39 83.23

IA-2A Enable 1.41 381 87.39 96.69 97.57 83.43

IA-2A RBA 1.58 380 78.70 98.67 98.91 75.13

IAA CRC 0.50 381 85.22 94.04 95.61 80.68

IAA Enable 0.41 381 82.61 94.70 95.96 78.14

IAA RBA −2.40 210 72.06 83.33 99.32 8.06

ZnT8A CRC −1.45 220 91.92 50.00 94.30 40.74

ZnT8A Enable 0.01 370 66.08 94.41 94.9. 63.68

ZnT8A RBA 4.95 204 79.29 100.00 100.00 12.77

***Supplemental Table 3.*** Sensitivity (Sens) and specificity (Spec) along with positive (ppv) and negative (npv) predictive values based on Chi^2^ (Table 3A) and Spec 98 (Table 3B) threshold and evaluated on the 90% remaining samples. One autoantibody (Total 1) is compared with two or more positive autoantibodies (Total 2) to stage type 1 diabetes in the two ADAP assays and RBA.

***Supplemental Table 3A.***

**Assay tp fp tn fn Sens Spec PPV NPV**

***Total 1***

CRC 2063 990 1253 7 1.00 0.56 0.68 0.99

Enable 2054 708 1536 16 0.99 0.68 0.74 0.99

RBA 2032 454 1789 38 0.98 0.80 0.82 0.98

***Total 2***

CRC 2002 360 1883 68 0.97 0.84 0.85 0.97

Enable 1971 131 2113 99 0.95 0.94 0.94 0.96

RBA 1918 69 2174 152 0.93 0.97 0.97 0.93

***Supplemental Table 3B.***

**Assay tp fp tn fn Sens Spec PPV NPV**

***Total 1***

CRC 2030 263 1980 40 0.98 0.88 0.89 0.98

Enable 2035 252 1992 35 0.98 0.89 0.89 0.98

RBA 1953 123 2120 117 0.94 0.95 0.94 0.95

***Total 2***

CRC 1828 30 2213 242 0.88 0.99 0.98 0.90

Enable 1905 27 2217 165 0.92 0.99 0.99 0.93

RBA 1475 11 2232 595 0.71 1.00 0.99 0.79

Abbreviations: tp is total positives, fp false positives, tn total negatives,

and fn false negatives

***Supplemental Table 4.*** The number of positive individuals/total number of analysed individuals, for each of the four antibodies as well as for the total positivity assessment with only one (Total 1) or two or more (Total 2) positive autoantibodies. Based on the 90% of individuals not included when optimizing the Chi^2^ and Spec98 threshold, respectively.

| **Sample** | **Assay** | **Metric** | **GADA** | **IA-2A** | **IAA** | **ZnT8A** | **Total 1** | **Total 2** |
| --- | --- | --- | --- | --- | --- | --- | --- | --- |
| ***PBC*** | CRC | Chi^2^ | 32/1336 (2.4%) | 125/1351 (9.3%) | 121/1350 (9.0%) | 91/200 (45.5%) | 297/1352 (22.0%) | 62/1352 (4.6%) |
|  | CRC | spec98 | 31/1336 (2.3%) | 39/1351 (2.9%) | 16/1350 (1.2%) | 0/200 (0.0%) | 80/1352 (5.9%) | 5/1352 (0.4%) |
|  | Enable | Chi^2^ | 128/1352 (9.5%) | 79/1352 (5.8%) | 68/1353 (5.0%) | 121/1295 (9.3%) | 329/1353 (24.3%) | 57/1353 (4.2%) |
|  | Enable | spec98 | 36/1352 (2.7%) | 46/1352 (3.4%) | 34/1353 (2.5%) | 33/1295 (2.5%) | 136/1353 (10.1%) | 11/1353 (0.8%) |
|  | RBA | Chi^2^ | 163/1352 (12.1%) | 60/1350 (4.4%) | 18/52 (34.6%) | 7/52 (13.5%) | 221/1352 (16.3%) | 27/1352 (2.0%) |
|  | RBA | spec98 | 59/1352 (4.4%) | 30/1350 (2.2%) | 0/52 (0.0%) | 0/52 (0.0%) | 84/1352 (6.2%) | 5/1352 (0.4%) |
| ***DOC*** | CRC | Chi^2^ | 30/410 (7.3%) | 77/410 (18.8%) | 158/410 (38.5%) | 275/410 (67.1%) | 336/410 (82.0%) | 153/410 (37.3%) |
|  | CRC | spec98 | 28/410 (6.8%) | 35/410 (8.5%) | 56/410 (13.7%) | 2/410 (0.5%) | 96/410 (23.4%) | 19/410 (4.6%) |
|  | Enable | Chi^2^ | 107/410 (26.1%) | 57/410 (13.9%) | 14/410 (3.4%) | 44/392 (11.2%) | 166/410 (40.5%) | 42/410 (10.2%) |
|  | Enable | spec98 | 29/410 (7.1%) | 37/410 (9.0%) | 9/410 (2.2%) | 8/392 (2.0%) | 61/410 (14.9%) | 13/410 (3.2%) |
|  | RBA | Chi^2^ | 25/410 (6.1%) | 8/410 (2.0%) | 99/410 (24.1%) | 66/410 (16.1%) | 153/410 (37.3%) | 33/410 (8.0%) |
|  | RBA | spec98 | 9/410 (2.2%) | 5/410 (1.2%) | 4/410 (1.0%) | 5/410 (1.2%) | 13/410 (3.2%) | 6/410 (1.5%) |
| **BDC** | CRC | Chi^2^ | 37/481 (7.7%) | 22/481 (4.6%) | 198/480 (41.2%) | 265/481 (55.1%) | 357/481 (74.2%) | 145/481 (30.1%) |
|  | CRC | spec98 | 36/481 (7.5%) | 3/481 (0.6%) | 51/480 (10.6%) | 3/481 (0.6%) | 87/481 (18.1%) | 6/481 (1.2%) |
|  | Enable | Chi^2^ | 179/481 (37.2%) | 13/481 (2.7%) | 26/481 (5.4%) | 31/460 (6.7%) | 213/481 (44.3%) | 32/481 (6.7%) |
|  | Enable | spec98 | 39/481 (8.1%) | 5/481 (1.0%) | 8/481 (1.7%) | 6/460 (1.3%) | 55/481 (11.4%) | 3/481 (0.6%) |
|  | RBA | Chi^2^ | 38/481 (7.9%) | 0/481 (0.0%) | 32/481 (6.7%) | 19/481 (4.0%) | 80/481 (16.6%) | 9/481 (1.9%) |
|  | RBA | spec98 | 23/481 (4.8%) | 0/481 (0.0%) | 3/481 (0.6%) | 0/481 (0.0%) | 26/481 (5.4%) | 0/481 (0.0%) |
| **BDD** | CRC | Chi^2^ | 1875/2068 (90.7%) | 1716/2070 (82.9%) | 1776/2070 (85.8%) | 1619/1821 (88.9%) | 2063/2070 (99.7%) | 2002/2070 (96.7%) |
|  | CRC | spec98 | 1868/2068 (90.3%) | 1551/2070 (74.9%) | 1565/2070 (75.6%) | 347/1821 (19.1%) | 2030/2070 (98.1%) | 1828/2070 (88.3%) |
|  | Enable | Chi^2^ | 1975/2070 (95.4%) | 1722/2070 (83.2%) | 1659/2070 (80.1%) | 1426/2054 (69.4%) | 2054/2070 (99.2%) | 1971/2070 (95.2%) |
|  | Enable | spec98 | 1876/2070 (90.6%) | 1652/2070 (79.8%) | 1546/2070 (74.7%) | 1116/2054 (54.3%) | 2035/2070 (98.3%) | 1905/2070 (92.0%) |
|  | RBA | Chi^2^ | 1699/2070 (82.1%) | 1653/2070 (79.9%) | 1407/1853 (75.9%) | 1449/1823 (79.5%) | 2032/2070 (98.2%) | 1918/2070 (92.7%) |
|  | RBA | spec98 | 1447/2070 (69.9%) | 1634/2070 (78.9%) | 446/1853 (24.1%) | 765/1823 (42.0%) | 1953/2070 (94.3%) | 1475/2070 (71.3%) |

***Supplemental Table 5*** Number of RBA measurements less than or equal to zero, per autoantibody and cohort.

| **Autoantibody** | **PBC** | **DOC** | **BDC** | **BDD** |
| --- | --- | --- | --- | --- |
| GADA | 1502 (1) | 456 (2) | 535 (0) | 2300 (0) |
| IA-2A | 1500 (144) | 456 (184) | 535 (1) | 2300 (66) |
| IAA | 58 (5) | 456 (76) | 535 (341) | 2057 (247) |
| ZnT8A | 58 (0) | 456 (0) | 535 (0) | 2021 (2) |

GADA (n0/n) 3/4793, p=0.0006; IA-2A 395/4791, p=0.08; IAA 669/3106, p=0.21; ZnT8A 2/3070, p=0.0007.

***Supplemental Table 6.*** The number of autoantibodies (npos) in the different assays and cohorts using spec 98 threshold.

**npos PBC DOC BDC BDD**

CRC

0 1272 314 394 40

1 75 77 81 202

2 4 15 6 584

3 1 2 NA 1015

4 NA 2 NA 229

Enable

0 1217 349 426 35

1 125 48 52 130

2 10 7 3 391

4 1 3 NA 736

3 NA 3 NA 778

RBA

0 1268 397 455 117

1 79 7 26 478

2 5 2 NA 736

3 NA 4 NA 614

4 NA NA NA 12

***Supplemental Table 7***. Sensitivity (Sens) and specificity (Spec) along with positive (ppv) and negative (npv) predictive values based on Chi2 and Spec 98 thresholds comparing children (PBC) and adult (BDC) controls with two or more positive autoantibodies for stage 1 type 1 diabetes in the two ADAP assays and in the RBA.

| **Controls** | **Assay** | **tp** | **fp** | **tn** | **fn** | **Sens** | **Spec** | **ppv** | **npv** |
| --- | --- | --- | --- | --- | --- | --- | --- | --- | --- |
| **Chi^2^** |  |  |  |  |  |  |  |  |  |
| PBC | CRC | 2002 | 360 | 1883 | 68 | 0.97 | 0.84 | 0.85 | 0.97 |
| BDC | CRC | 1953 | 155 | 2088 | 117 | 0.94 | 0.93 | 0.93 | 0.95 |
| PBC | Enable | 1971 | 131 | 2113 | 99 | 0.95 | 0.94 | 0.94 | 0.96 |
| BDC | Enable | 2000 | 221 | 2023 | 70 | 0.97 | 0.90 | 0.90 | 0.97 |
| PBC | RBA | 1918 | 69 | 2174 | 152 | 0.93 | 0.97 | 0.97 | 0.94 |
| BDC | RBA | 2008 | 383 | 1860 | 62 | 0.97 | 0.83 | 0.84 | 0.97 |
| **Spec98** |  |  |  |  |  |  |  |  |  |
| PBC | CRC | 1828 | 30 | 2213 | 242 | 0.88 | 0.99 | 0.98 | 0.90 |
| BDC | CRC | 1194 | 5 | 2238 | 876 | 0.58 | 0.99 | 0.99 | 0.72 |
| PBC | Enable | 1905 | 27 | 2217 | 165 | 0.92 | 0.99 | 0.99 | 0.93 |
| BDC | Enable | 1564 | 7 | 2237 | 506 | 0.76 | 0.99 | 0.99 | 0.82 |
| PBC | RBA | 1475 | 11 | 2232 | 595 | 0.71 | 0.99 | 0.99 | 0.79 |
| BDC | RBA | 1367 | 6 | 2237 | 703 | 0.66 | 0.99 | 0.99 | 0.76 |

Abbreviations: tp is total positives, fp false positives, tn total negatives and fn false negatives.
