# Supplementary figures and images for: Childhood screening for type 1 diabetes comparing automated multiplex Antibody Detection by Agglutination-PCR (ADAP) with single plex islet autoantibody radiobinding assays
Source: eBioMedicine. 2024 May 8;104:105144. doi: 10.1016/j.ebiom.2024.105144 (PMC11090024; doi:10.1016/j.ebiom.2024.105144)

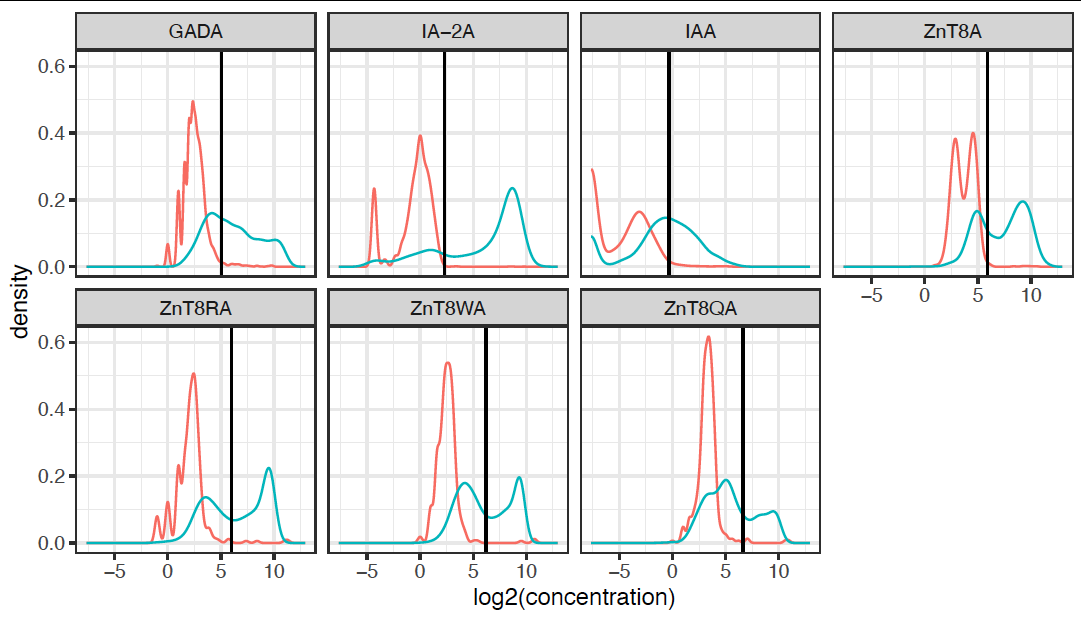


Supplemental Figure 1

Supplement: Supplement Figure S1 [file mmc3.docx]

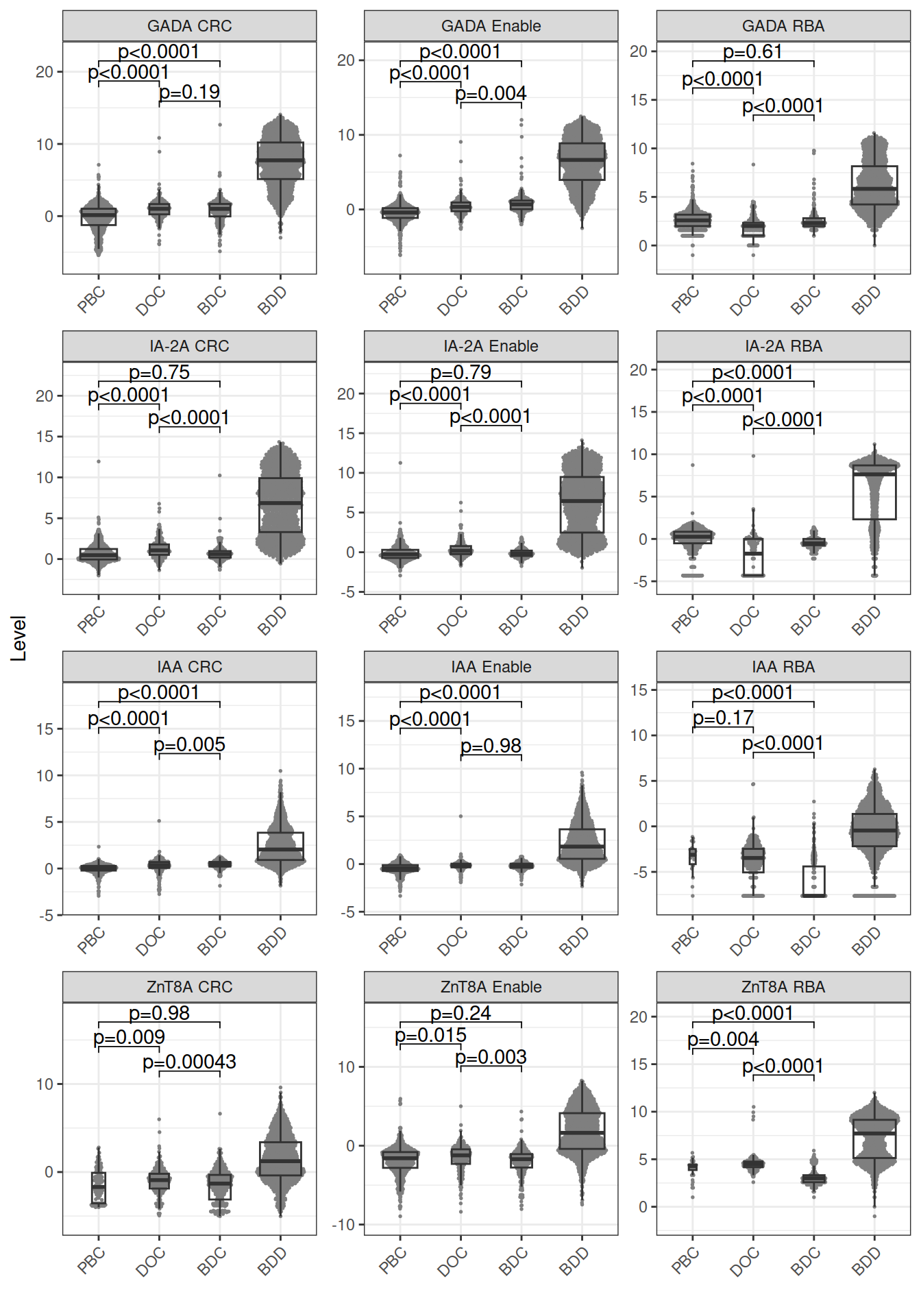


Supplemental Figure 2

Supplement: Supplement Figure S2 [file mmc4.docx]

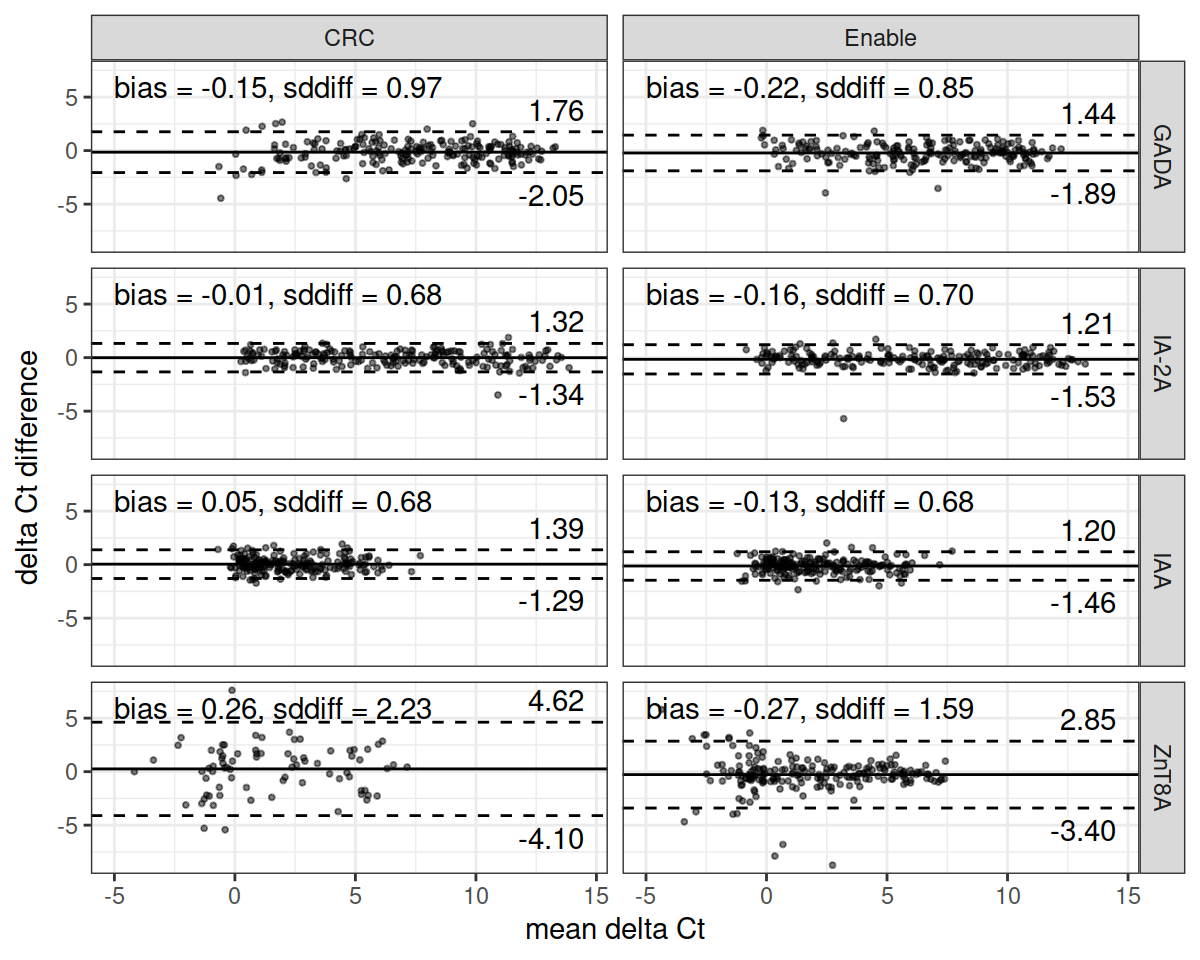


Supplemental Figure 3.

Supplement: Supplement Figure S3 [file mmc5.docx]
